# Aberrant expression of the microtubule-associated protein tau is an independent prognostic feature in prostate cancer

**DOI:** 10.1186/s12885-019-5390-1

**Published:** 2019-03-01

**Authors:** Cornelia Schroeder, Jan Grell, Claudia Hube-Magg, Martina Kluth, Dagmar Lang, Ronald Simon, Doris Höflmayer, Sarah Minner, Eike Burandt, Till S. Clauditz, Franziska Büscheck, Frank Jacobsen, Hartwig Huland, Markus Graefen, Thorsten Schlomm, Guido Sauter, Stefan Steurer

**Affiliations:** 1 Institute of Pathology, University Medical Center Hamburg-Eppendorf, Martinistrasse 52, D-20246 Hamburg, Germany; 2 General, Visceral and Thoracic Surgery Department and Clinic, University Medical Center Hamburg-Eppendorf, Martinistrasse 52, D-20246 Hamburg, Germany; 3 Martini-Clinic, Prostate Cancer Center, University Medical Center Hamburg-Eppendorf, Martinistrasse 52, D-20246 Hamburg, Germany; 4 Department of Urology, Charité - Universitätsmedizin Berlin, Charitéplatz 1, D-10117 Berlin, Germany

**Keywords:** MAPT, Tau, ERG, PTEN, Deletion, Prostate cancer

## Abstract

**Background:**

Microtubule-associated protein Tau (MAPT) overexpression has been linked to poor prognosis and decreased response to taxane-based therapies in several cancer types, but its relevance in prostate cancer is unknown.

**Methods:**

In this study, MAPT expression was analyzed by immunohistochemistry on a tissue microarray containing 17,747 prostate cancers.

**Results:**

MAPT was absent in normal prostate epithelial cells but detectable in 1004 (8.2%) of 12,313 interpretable cancers. Its expression was associated with advanced tumor stage, high Gleason grade, positive lymph nodes, and early biochemical recurrence (*p* < 0.0001 each). For example, MAPT was found in 3.6% of 2072 Gleason ≤3 + 3 cancers but in 14.4% of 704 Gleason ≥4 + 4 cancers. High-level MAPT staining was also linked to *TMPRSS2:ERG* fusions (*p* < 0.0001). MAPT staining was seen in 15.2 and 16% of cancers with *TMPRSS2:ERG* fusion detected by immunohistochemistry and fluorescence in-situ hybridization, but in only 3.5 and 3.9% of cancers without ERG staining or *ERG* rearrangements. Moreover, an association was found between MAPT expression and *PTEN* deletions, with 19% MAPT positivity in 948 *PTEN* deleted cancers but only 7% MAPT positivity in 3895 tumors with normal *PTEN* copy numbers (*p* < 0.0001). Multivariate analysis revealed that the prognostic value of MAPT was independent from established parameters. Conventional large section analyses showed intratumoral MAPT heterogeneity in all three analyzed cancers.

**Conclusions:**

The results of our study identify MAPT, as a moderate prognostic marker in prostate cancer, whose clinical impact, however, may be limited due to the rarity and heterogeneity of its expression.

**Electronic supplementary material:**

The online version of this article (10.1186/s12885-019-5390-1) contains supplementary material, which is available to authorized users.

## Background

In men with Western lifestyle the most prevalent cancer is prostate cancer [1]. Although most cancers show an indolent course, the disease still represents the third most common cause of cancer related death in men. Therefore a specific and sensitive prediction of aggressive forms is warranted to improve decision-making [2, 3]. At present Gleason grade and tumor extent on biopsies, preoperative prostate-specific antigen (PSA), and clinical stage are established pretreatment prognostic parameters. These parameters are statistically powerful but not sufficiently reliable for optimal individual outcome prediction. For example the Gleason grade suffers from substantial interobserver variation [4]. Therefore the identification of new clinically applicable molecular markers may enable a more reliable prediction of prostate cancer aggressiveness in the future.

MAPT facilitates tubulin assembly and microtubule stabilization [5]. MAPT is mainly expressed in neuronal axons and glial cell cytoplasm, but is also present in various non-neuronal cells including lymphocytes, epithelial and glandular cells [5–8]. Aberrant expression of MAPT has been reported for many cancer types such as gastric, breast, and colorectal cancer [9–12], and has been linked to adverse tumor features and poor prognosis in some of them [12]. Little is known about the role of MAPT in prostate cancer. Only a few studies demonstrated MAPT expression in prostate cancer cell lines and in clinical samples but did not attempt to link MAPT expression to clinical features of the disease [13–15]. However, MAPT might be of interest in prostate cancer since overexpression has been found to represent a prognostic marker in several cancers [12, 16, 17]. MAPT overexpression has also been linked to resistance to taxane-based therapies in various other cancer types [10–12, 18]. To date, taxanes are the most important cytotoxic agents for advanced and hormone-refractory prostate cancer [19–21].

Here, we employed a large - more than 17,000 prostate cancers - and highly annotated tissue microarray (TMA) to elucidate the role of MAPT expression in this disease.

## Methods

### Patients

The 17,747 patients had radical prostatectomy between 1992 and 2014 at the University Medical Center Hamburg-Eppendorf (Department of Urology and the Martini Clinics). Follow-up was available for 14,464 patients with a median follow-up of 48 months (range: 1 to 275 months; Table S1). PSA recurrence was defined as a postoperative PSA of 0.2 ng/ml and increasing in subsequent measurements. Histological analysis was done by a standard method [22]. Quantitative Gleason grading was performed using the percentage of Gleason 4 and tertiary Gleason 5 patterns as described before [23]. The TMA spot size was 0.6 mm and each TMA had internal controls with normal prostate tissue [24, 25]. The highly annotated TMA contained data on ERG expression [26], *ERG* break apart fluorescence in situ hybridization (FISH) [27] and deletion status of 5q21 (*CHD1*) [28], 6q15 (*MAP3K7*) [29], 10q23 (*PTEN*) [30]) and 3p13 (*FOXP1*) [31]) cancers.

### Immunohistochemistry (IHC)

**Fig. 1:**
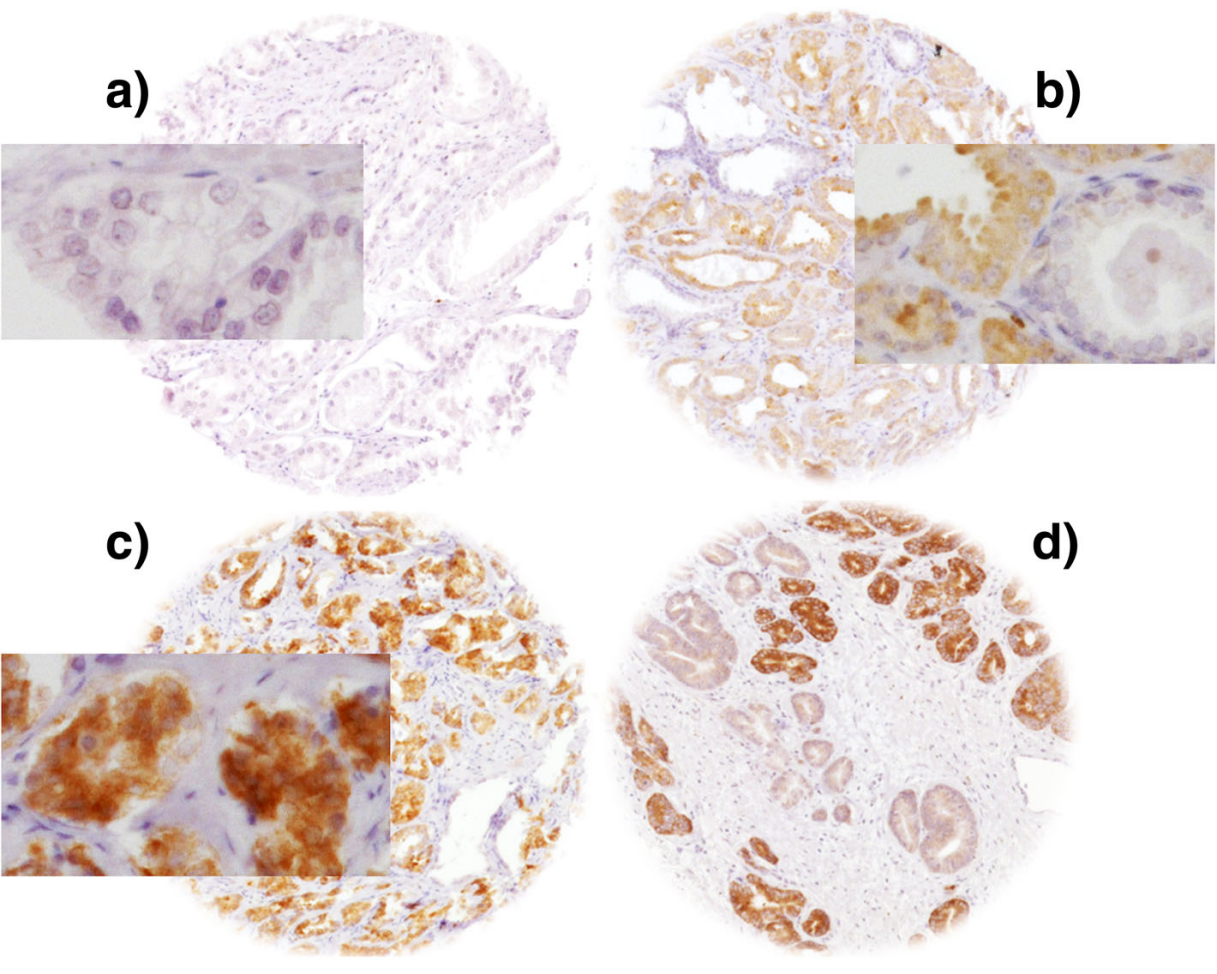
Representative images of (**a**) negative, (**b**) low, (**c**) high and (**d**) heterogeneous microtubule-associated protein Tau (MAPT) staining in prostate cancer at 100x and 400x (inset) magnification; original spot size was 600 μm

Freshly cut TMA sections were stained the same day and in one experiment. Slides were deparaffinized and exposed to heat-induced antigen retrieval for 5 min in an autoclave at 121 °C in pH 7.8 Tris-EDTA-citrate buffer. Primary antibody specific for MAPT (mouse monoclonal antibody, clone 2B2.100, Biomol GmbH, Germany; cat#T1029; dilution 1:450) was applied at 37 °C for 60 min. Bound antibody was visualized with the EnVision Kit (Dako, Glostrup, Denmark) according to the manufacturer’s directions. MAPT staining was found in the cytoplasm of cells. In MAPT positive cancers, staining was mostly seen in all (100%) tumor cells. Accordingly, the average staining intensity in prostate cancer cells was recorded in three categories as negative (no detectable staining), low and high staining (Fig. 1).

### Statistical analysis

Contingency tables and the chi-square test were computed to study association between MAPT expression and clinico-pathological variables. Kaplan-Meier analysis and the log-rank test were employed with PSA recurrence as the endpoint. Cox proportional hazards were calculated in a uni- and a multivariate model to test for independence and significance of the variables. JMP 12 (SAS Institute Inc., NC, USA) was used.

## Results

A total of 12,313 (69%) of tumor samples were interpretable. Reason for non-informative cases (5434 spots; 31%) included lack of tissue samples or absence of unequivocal cancer tissue in the TMA spot. Normal prostate tissues showed no staining. In tumors, MAPT staining was seen in 8.2% (1004 / 12,313) samples and was considered low in 7.1% and high in 1.1% of cancers. Typical pictures of MAPT immunostaining are given in Fig. 1. Because heterogeneous findings were occasionally seen in TMA spots (Fig. 1d), three cancers with high MAPT expression were selected for analysis of intratumoral heterogeneity. In these cases, additional IHC analysis was done on conventional large sections of all available tumor-containing tissue blocks. Ten slides per cancer were analyzed. All cancers showed distinct areas with and without MAPT staining.

### Association with *TMPRSS2:ERG* fusion status and ERG protein expression

MAPT staining and *TMPRSS2:ERG* fusion status by FISH were available from 5028 and by IHC from 7500 cases. In 96% (4644/4849) of the cases ERG FISH and IHC results were concordant. MAPT staining was linked to *TMPRSS2:ERG* rearrangement and ERG positivity (Additional file 1: Figure S1).

### Association with tumor phenotype and PSA recurrence

**Fig. 2:**
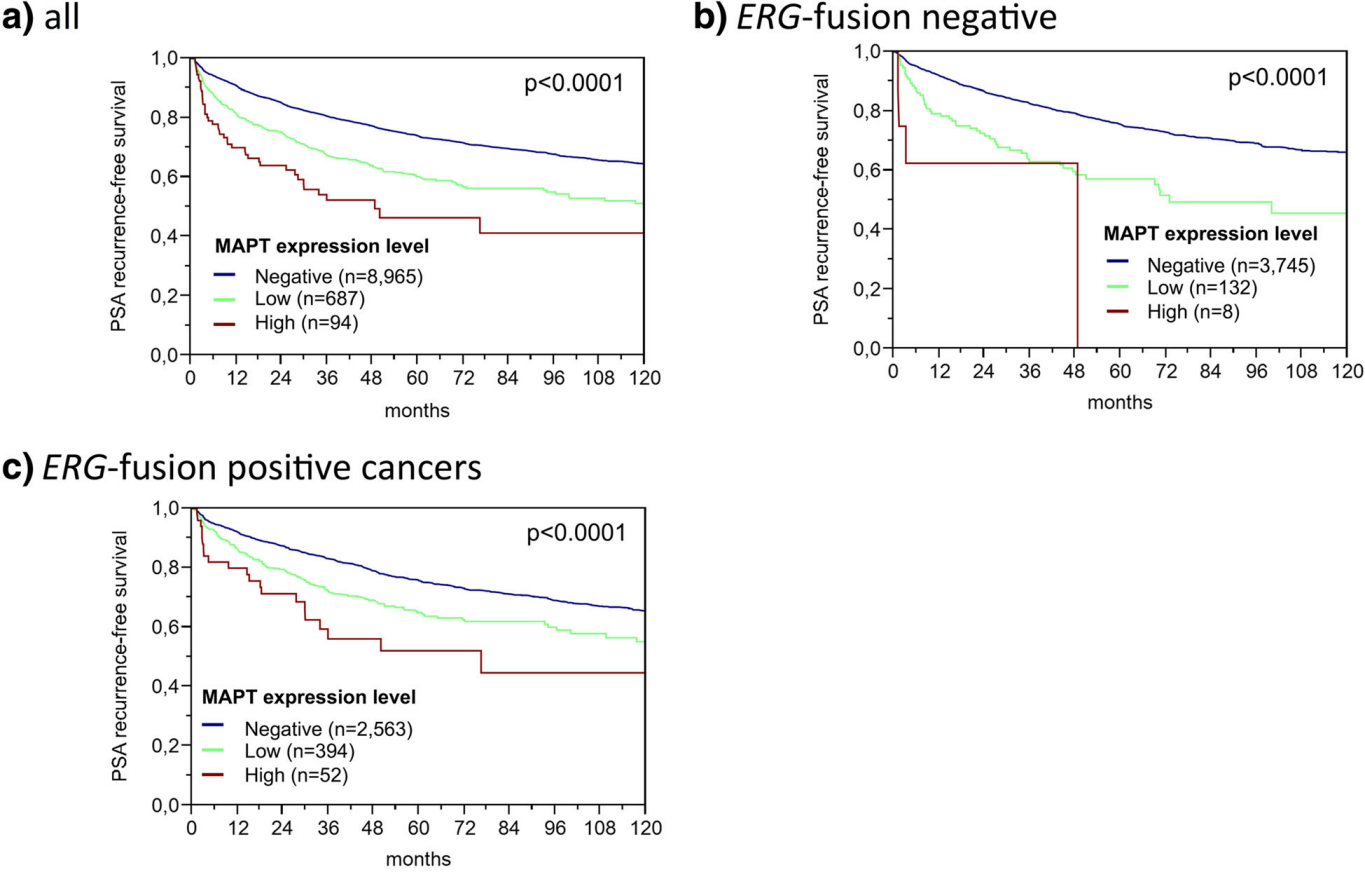
Association between microtubule-associated protein Tau (MAPT) expression and prostate specific antigen (PSA) recurrence in (**a**) all cancers, (**b**) the *TMPRSS2:ERG* negative and (**c**) the *TMPRSS2:ERG* positive subset

**Fig. 3:**
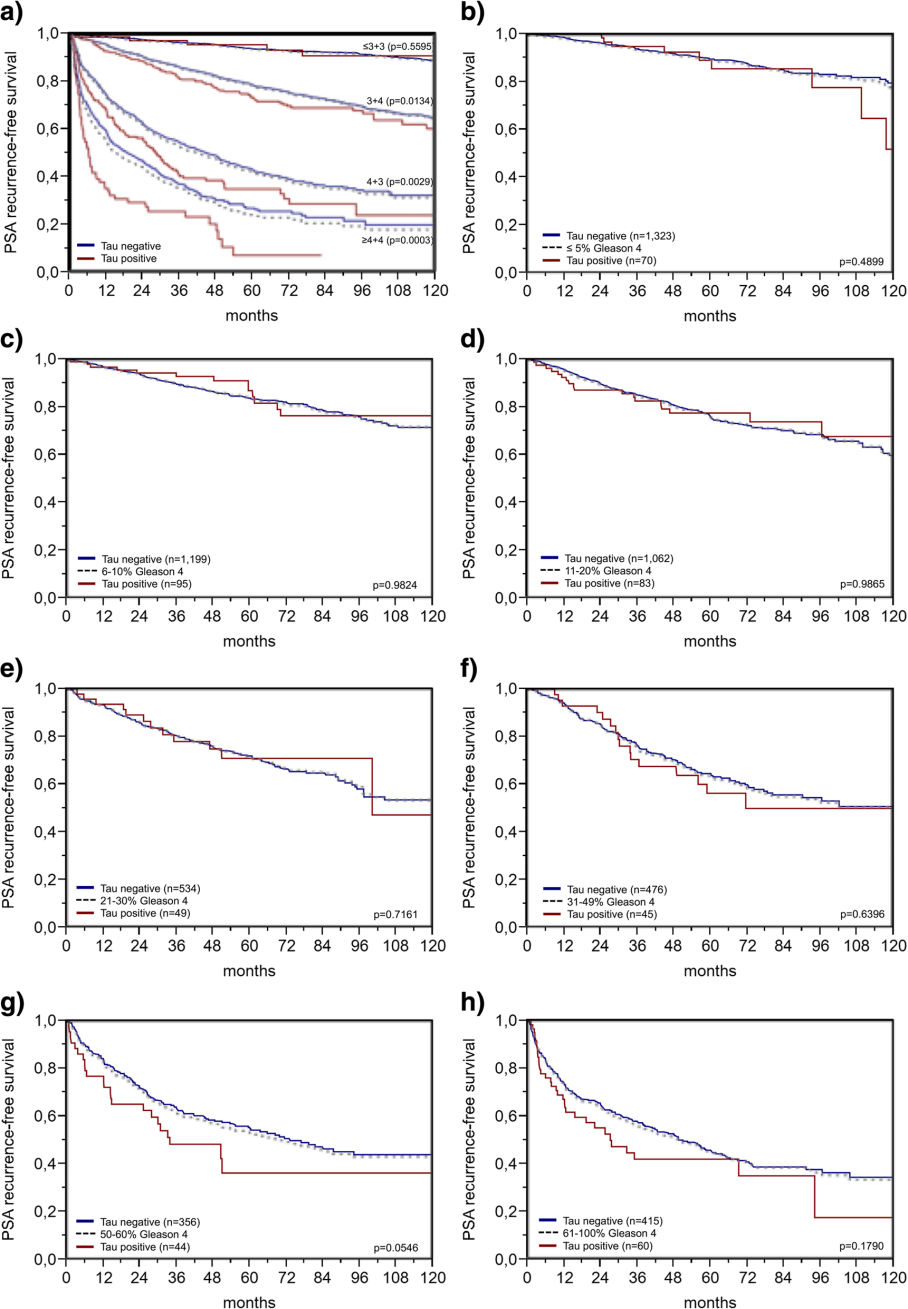
Kaplan-Meier plots of prostate specific antigen (PSA) recurrence after radical prostatectomy and negative or positive (low and high) microtubule-associated protein Tau (MAPT) expression in subsets defined by (**a**) classical and (**b-h**) quantitative Gleason score, defined by the percentage of Gleason 4 grade

MAPT expression levels were significantly associated with advanced tumor stage, high Gleason grade, positive nodal stage, and positive resection margin (*p* ≤ 0.0011 each, Table 1). These associations held also true in the subsets of ERG negative and ERG positive cancers, although not all *p* values remained significant probably due to the overall small numbers of MAPT positive cancers (Additional file 1: Table S2 and S3). High MAPT expression levels were also associated with a higher risk for biochemical recurrence in all cancers and in the subsets of ERG positive and ERG negative cancers (*p* < 0.0001 each, Fig. 2). To further validate the prognostic power of MAPT, we tested within subsets of identical classical and quantitative Gleason score. In line with the Cox hazard ratio analysis (Additional file 1: Table S4), MAPT staining provided prognostic information beyond the Gleason score in subsets defined by an identical traditional Gleason score (Fig. 3a) and in the subgroup with 50–60% Gleason 4 pattern (Fig. 3g) defined by the quantitative Gleason score (Fig. 3b-h).

**Table 1:** Association between microtubule-associated protein Tau (MAPT) staining and prostate cancer phenotype

		MAPT (%)			
Parameter	N	Negative	Low	High	*P*
All cancers	12,313	91.8	7.1	1.1	
Tumor stage					
pT2	7764	94.2	5.2	0.6	< 0.0001
pT3a	2809	88.9	9.6	1.5	
pT3b-pT4	1688	85.7	11.7	2.6	
Gleason grade					
≤ 3 + 3	2072	96.4	3.3	0.3	< 0.0001
3 + 4	6702	92.6	6.7	0.8	
3 + 4 Tertiary 5	614	91.0	8.1	0.8	
4 + 3	1257	88.5	9.7	1.8	
4 + 3 Tertiary 5	925	86.3	10.9	2.8	
≥ 4 + 4	704	85.5	11.6	2.8	
Lymph node metastasis					
N0	7604	91.3	7.5	1.2	< 0.0001
N+	943	85.9	11.7	2.4	
Preoperative PSA level (ng/ml)					
< 4	1418	89.6	8.9	1.6	0.0506
4–10	7278	92.3	6.7	1.0	
10–20	2629	91.8	7.2	1.0	
> 20	918	91.8	7.3	0.9	
Surgical margin					
Negative	9733	92.3	6.7	1.0	0.0011
Positive	2536	90.0	8.6	1.3

### Association with other key genomic deletions

**Fig. 4:**
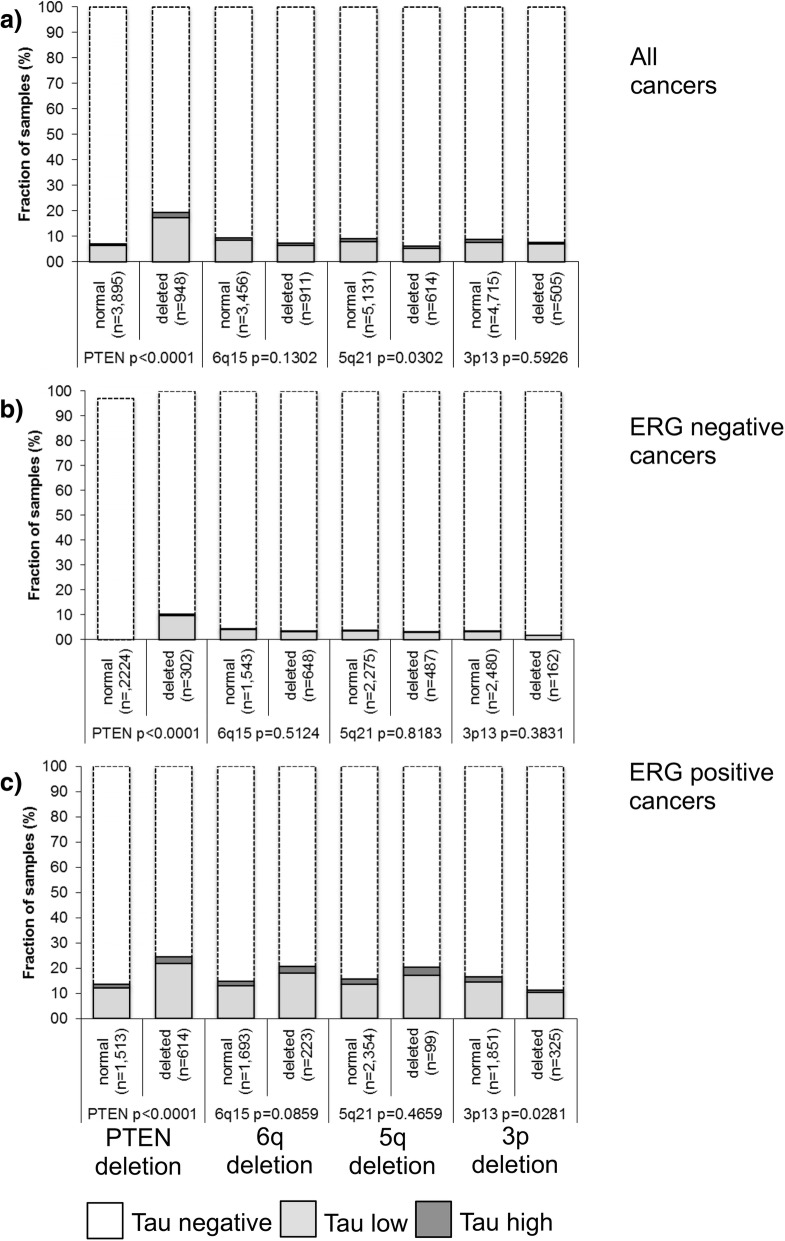
Association between positive microtubule-associated protein Tau (MAPT) staining and 10q23 (*PTEN*), 5q21 (*CHD1*), 6q15 (*MAP3K7*), 3p13 (*FOXP1*) deletions in (**a**) all cancers, (**b**) the *TMPRSS2:ERG* negative and (**c**) the *TMPRSS2:ERG* positive subset

Previous studies showed that prostate cancers could be grouped by various somatic mutations including *TMPRSS2:ERG* fusions and *PTEN,* 3p13, 5q21 and 6q15 genomic deletions. These alterations are of interest because they are linked to poor prognosis and either to the ERG-fusion positive (PTEN, 3p) or the ERG-fusion negative subset (5q, 6q). A comparison of MAPT expression levels with these deletions revealed a significant association between high MAPT expression and *PTEN* deletions irrespectively of the ERG status (*p* < 0.0001, Fig. 4). MAPT expression was largely unrelated to other deletions.

### Multivariate analysis

Four different scenarios were performed evaluating the clinical relevance of MAPT expression (Table 2). For example the preoperative scenario 4 included the Gleason grade obtained on the original biopsy, the PSA level, the cT stage and the MAPT expression. MAPT proved to be an independent prognostic parameter in all four scenarios when all tumors were analyzed (*p* < 0.0001 each, Table 2). This held also true for ERG negative and ERG positive cancers (*p* ≤ 0.02, Table 2). The hazard ratios for PSA recurrence-free survival after prostatectomy for the univariate and multivariate model of the established preoperative prognostic parameters and MAPT expression (scenario 4) are shown in the Table S4. MAPT expression was an equally strong prognostic marker as the other known preoperative prognostic markers in both analyses.

**Table 2:** Multivariate analyses including microtubule-associated protein Tau (MAPT) expression in all cancers, the *ERG* negative and *ERG* positive subset

			P for PSA recurrence-free survival after prostatectomy							
Subset	Scenario^a^	N	Preoperative PSA-level	pT-stage	cT-stage	Gleason prostatectomy	Gleason biopsy	pN-stage	R-status	MAPT-expression
All cancers										
	1	6467	< 0.0001	< 0.0001	–	< 0.0001	–	< 0.0001	< 0.0001	< 0.0001
	2	9690	< 0.0001	< 0.0001	–	< 0.0001	–	–	< 0.0001	< 0.0001
	3	9545	< 0.0001	–	< 0.0001	< 0.0001	–	–	–	< 0.0001
	4	8146	< 0.0001	–	< 0.0001	–	< 0.0001	–	–	< 0.0001
ERG-negative										
	1	2541	0.0215	< 0.0001	–	< 0.0001	–	0.0005	0.0601	0.0195
	2	3873	0.0013	< 0.0001	–	< 0.0001	–	–	< 0.0001	0.0180
	3	3833	< 0.0001	–	< 0.0001	< 0.0001	–	–	–	0.0013
	4	3779	< 0.0001	–	< 0.0001	–	< 0.0001	–	–	< 0.0001
ERG-positive										
	1	1900	0.0005	< 0.0001	–	< 0.0001	–	0.0145	0.0010	0.0122
	2	2995	< 0.0001	< 0.0001	–	< 0.0001	–	–	< 0.0001	0.0185
	3	2944	< 0.0001	–	< 0.0001	< 0.0001	–	–	–	0.0115
	4	2900	< 0.0001	–	< 0.0001	–	< 0.0001	–	–	0.0003

^a^Scenario 4 combines preoperatively available parameters (preoperative PSA, clinical tumor (cT) stage, and Gleason grade obtained on the original biopsy) with the postoperative MAPT expression. In scenario 3 the biopsy Gleason is replaced by the Gleason grade obtained on radical prostatectomy. In scenario 2 cT stage is superseeded by pathological tumor (pT) stage and surgical margin (R) status. In scenario 1 the lymph node (pN) stage is added

## Discussion

The results of our study identify MAPT overexpression as a moderate prognostic feature occurring in a relatively small subset of prostate cancers.

In this study, detectable MAPT expression was seen in about 8% of prostate cancers whereas normal prostate tissues remained negative under the selected experimental conditions. Only one study has analyzed MAPT expression by IHC in prostate cancer before. Cirak et al. reported 23% MAPT positive cases in a series of 30 prostate cancers [13]. It is well possible, that the large section approach of Cirak et al. lead to a higher detection rate of tumors with a heterogeneous MAPT expression. Our data indeed suggest that MAPT expression might be heterogeneous in a considerable fraction of tumors. Clear-cut heterogeneity was even found in some TMA spots (Fig. 1d) and a thorough analysis of all cancer-containing tissue blocks of three of our cancers with high MAPT expression on TMA spots always revealed both MAPT positive and MAPT negative cancer areas. Such heterogeneity represents a limitation for TMA studies analyzing only single spots per tumor.

MAPT overexpression was associated with to unfavorable tumor phenotype and early biochemical recurrence in this study (*p* < 0.0001 each). The independent prognostic impact of MAPT overexpression from established prognostic parameters and the difference in the five-year recurrence rate of more than 20% between patients with and without detectable MAPT expression argues for a potential clinical relevance of this molecular feature. A similarly strong prognostic role has recently been described for aberrant βIII-tubulin (TUBB3) expression in prostate cancer. TUBB3 is a microtubule protein, which is normally expressed in cells of neuronal origin but not in prostate epithelium [32]. Overall, the striking prognostic impact of the expression of proteins influencing structure and maintenance of microtubules suggest a considerable impact of composition and function of the cytoskeleton on the behavior of cancer cells.

The extensive molecular database attached to our TMA allowed us to further study the role of MAPT expression in prostate cancer and to search for possible interactions. About 50% of prostate cancers carry gene fusions linking the androgen-regulated *TMPRSS2* with the transcription factor *ERG* [26, 33]. As a result of this rearrangement, *ERG* becomes androgen regulated and massively overexpressed. Our data demonstrate strikingly higher MAPT expression levels in ERG positive than in ERG negative cancers. This finding is consistent with data suggesting that ERG may have a regulatory role in microtubule dynamics [17, 34] and that ERG can even destabilize microtubules by binding soluble tubulin in the cytoplasm [35]. The exact molecular mechanism for this is unknown. According to the eukaryotic promoter database [36] MAPT is not a direct target of the *ERG* transcription factor. It is possible, however, that ERG has an indirect impact on MAPT transcription through at least one of its more than 1600 target genes [37–39]. Our comparison of MAPT expression with frequent genomic deletions identified *PTEN* as the only deletion linked to high MAPT expression. This fits well to earlier work in neurodegenerative diseases reporting that *PTEN* can affect MAPT phosphorylation, aggregation or it’s binding to microtubules [40, 41].

The existing data suggest a general role of MAPT protein in cancer. High rates of MAPT positivity have been reported from several other important cancer types including 43–52% in breast cancer [16, 42, 43], 63–74% in ovarian cancer [12, 44], and 55–70% in gastric cancer [11, 45, 46]. The clinical and prognostic value of MAPT may greatly depend on the tumor type. For example, high MAPT protein expression level has been linked to good prognosis in breast cancer [47], but to poor prognosis in ovarian cancer [12]. It is unknown why MAPT exerts a different impact on tumor cell aggressiveness in different cancer types. As the microtubule composition varies between cell types, it may be speculated that MAPT induced modifications of the microtubule dynamics may have a diverse impact on cell behavior depending on the tissue of origin. It is also known that MAPT interacts with other cancer related proteins and pathways. For example, it has been shown that MAPT can cooperate with various growth related kinases such PI3K, Fyn, cSrc, and Fgr [14, 48, 49]. Such kinases may have a different role in different cell types. Moreover, MAPT interactions depend on its phosphorylation status. Substantial differences in cell lines derived from prostate and brain cancers suggest that MAPT phosphorylation might strongly depend on the tumor type [14, 50–52].

In several tumor types, MAPT has been suggested to represent a potential predictive marker in patients treated with taxanes [12, 46, 53–57]. MAPT competes with taxanes for the same binding site at the microtubules. Although MAPT stabilizes microtubules in the same way as paclitaxel, its binding is more reversible [18]. Consequently, overexpression of MAPT has been suggested to render microtubules insensitive to paclitaxel therapy [18, 58, 59]. In prostate cancer, taxanes are the most important cytotoxic agents for advanced metastatic disease. However, response rates in clinical studies (measured as a 50% decline of PSA) are about 45–50% [60]. It would be interesting to study the relationship between expression of proteins related to the microtubules system - such as MAPT and TUBB3 - and response to taxanes in prostate cancer in clinical trials.

## Conclusions

MAPT expression is a moderate and independent prognostic factor in prostate cancer, which is particularly linked to *PTEN*-deleted cancers. Heterogeneity of expression within tumors may limit the practical use of MAPT measurement in clinical practice, however.

## Additional file

Additional file 1: **Table S1.** Pathological and clinical data of the arrayed prostate cancers. **Table S2.** Association between microtubule-associated protein Tau (MAPT) staining results and prostate cancer phenotype in *ERG* fusion *negative* tumors. **Table S3.** Association between microtubule-associated protein Tau (MAPT) staining results and prostate cancer phenotype in *ERG* fusion *positive* tumors. **Table S4.** Cox proportional hazards for PSA recurrence-free survival after prostatectomy of established preoperative prognostic parameter and MAPT expression. **Figure S1.** Association between positive microtubule-associated protein Tau (MAPT) staining and ERG status (IHC/FISH) in all cancers. (PDF 231 kb)

## Abbreviations

CHD1: Chromodomain-Helicase-DNA-Binding Protein 1
FISH: Fluorescence in-situ hybridization
FOXP1: Forkhead box protein P1
IHC: Immunohistochemistry
MAP3K7: Mitogen-Activated Protein Kinase Kinase Kinase 7
MAPT: Microtubule-associated protein Tau
PSA: Prostate specific antigen
PTEN: Phosphatase and tensin homolog
RPE: Radical prostatectomy
TMA: Tissue microarray
TMPRSS2:ERG: Transmembrane protease, serine 2: ETS-related gene fusion
TUBB3: Tubulin beta 3
