# Supplementary material for: Aberrant expression of the microtubule-associated protein tau is an independent prognostic feature in prostate cancer
Source: BMC Cancer. 2019 Mar 1;19:193. doi: 10.1186/s12885-019-5390-1 (PMC6397474; doi:10.1186/s12885-019-5390-1)
Supplement: Supplementary file 1 — Table S1. Pathological and clinical data of the arrayed prostate cancers. Table S2. Association between microtubule-associated protein Tau (MAPT) staining results and prostate cancer phenotype in ERG fusion negative tumors. Table S3. Association between microtubule-associated protein Tau (MAPT) staining results and prostate cancer phenotype in ERG fusion positive tumors. Table S4. Cox proportional hazards for PSA recurrence-free survival after prostatectomy of established preoperative prognostic parameter and MAPT expression. Figure S1. Association between positive microtubule-associated protein Tau (MAPT) staining and ERG status (IHC/FISH) in all cancers. (PDF 231 kb) [file 12885_2019_5390_MOESM1_ESM.pdf]

Table S1 Pathological and clinical data of the arrayed prostate cancers

	**Study cohort on TMA (n= 17 747)**	**Biochemical relapse among category**
**Follow-up**	14 464	3 612 (25%)
Mean/ median (month)	56.3 / 48.0	-
**Age (y)**		
≤50	433	66 (15%)
51-59	4 341	839 (19%)
60-69	9 977	2 073 (21%)
≥70	2 936	634 (22%)
**Pretreatment PSA (ng/ml)**		
<4	2 225	313 (14%)
4-10	10 520	1 696 (16%)
10-20	3 662	1 043 (29%)
>20	1 231	545 (44%)
**pT stage (AJCC 2002)**		
pT2	11 518	1 212 (11%)
pT3a	3 842	1 121 (29%)
pT3b	2 233	1 213 (54%)
pT4	85	63 (74%)
**Gleason grade**		
≤3+3	3 570	264 (7%)
3+4	9 336	1 436 (15%)
3+4 Tertiary 5	1 697	165 (10%)
4+3	2 903	683 (24%)
4+3 Tertiary 5	1 187	487 (41%)
≥4+4	999	531 (53%)
**Nodal (pN) stage**		
pN0	10 636	2 243 (21%)
pN+	1 255	700 (56%)
**Surgical margin (R) status**		
Negative	14 297	2 307 (16%)
Positive	3 388	1 304 (39%)

NOTE: Numbers do not always add up to 17 747 in the different categories because of cases with missing data. Abbreviation: AJCC, American Joint Committee on Cancer.

Table S2 Association between microtubule-associated protein Tau (MAPT) staining results and prostate cancer phenotype in *ERG* fusion *negative* tumors

		Tau			
**Parameter**	**n evaluable**	**negative (%)**	**low (%)**	**high (%)**	**p value**
**All cancers**	4,207	96.5	3.3	0.2	
**Tumor stage**					
pT2	2,792	97.9	1.9	0.1	<0.0001
pT3a	864	94.8	5.2	0.0	
pT3b-pT4	538	91.8	7.4	0.7	
**Gleason grade**					
≤3+3	794	98.5	1.4	0.1	
3+4	2,242	97.4	2.6	0.0	
3+4 Tert.5	199	95.0	5.0	0.0	<0.0001
4+3	465	94.0	5.8	0.2	
4+3 Tert.5	267	93.3	6.0	0.7	
≥4+4	237	91.6	7.2	1.3	
**Lymph node metastasis**					0.0719
N0	2,498	95.8	4.0	0.2	
N+	249	92.8	6.4	0.8	
**Preop. PSA level (ng/ml)**					
<4	418	93.5	5.7	0.7	0.0005
4-10	2,485	96.9	2.9	0.2	
10-20	933	97.5	2.5	0.0	
>20	347	94.2	5.8	0.0	
**Surgical margin**					0.3312
negative	3,321	96.7	3.1	0.2	
positive	874	95.7	4.1	0.2	

Table S3 Association between microtubule-associated protein Tau (MAPT) staining results and prostate cancer phenotype in *ERG* fusion *positive* tumors

		Tau			
**Parameter**	**n evaluable**	**negative (%)**	**low (%)**	**high (%)**	**p value**
**All cancers**	3,293	84.8	13.4	1.8	
**Tumor stage**					
pT2	1,950	87.7	11.2	1.1	<0.0001
pT3a	884	81.7	16.2	2.1	
pT3b-pT4	447	78.5	17.4	4.0	
**Gleason grade**					
≤3+3	644	93.0	6.4	0.6	
3+4	1,901	85.7	13.0	1.2	
3+4 Tert.5	102	75.5	21.6	2.9	<0.0001
4+3	323	77.1	19.5	3.4	
4+3 Tert.5	189	74.1	19.6	6.3	
≥4+4	131	73.3	22.9	3.8	
**Lymph node metastasis**					
N0	1,871	82.5	15.2	2.3	0.3988
N+	211	78.7	18.5	2.8	
**Preop. PSA level (ng/ml)**					
<4	451	83.1	14.4	2.4	0.2247
4-10	2,030	86.1	12.3	1.6	
10-20	589	81.8	16.1	2.0	
>20	198	84.8	13.6	1.5	
**Surgical margin**					
negative	2,548	85.6	12.8	1.7	0.114
positive	732	82.4	15.6	2.0	

Table S4 Cox proportional hazards for PSA recurrence-free survival after prostatectomy of established preoperative prognostic parameter and MAPT expression

Variable	Univariate analysis	Multivariate analysis
**Gleason grade biopsy**		
3+4 vs. ≤3+3	2.20 (2.00-2.41) ***	1.79 (1.59-2.00) ***
4+3 vs. 3+4	1.85 (1.66-2.05) ***	1.74 (1.54-1.97) ***
≥4+4 vs. 4+3	1.48 (1.32-1.66) ***	1.33 (1.17-1.53) ***
**cT-stage**		
T3a vs. T1c	2.16 (1.73-2.66) ***	1.49 (1.12-1.94) *
**Preoperative PSA-level (ng/µl)**		
4-10 vs. <4	1.26 (1.12-1.42) **	1.33 (1.13-1.57) **
10-20 vs. 4-10	2.08 (1.93-2.25) ***	1.58 (1.42-1.75) ***
>20 vs. 10-20	1.95 (1.76-2.16)***	1.63 (1.42-1.86) ***
**MAPT expression**		
Positive vs. negative	1.78 (1.58-2.01) ***	1.49 (1.29-1.70) ***
Strong vs. negative	2.68 (1.94-3.57)***	-

Confidence interval (95%) in brackets; asterisk indicate significance level: * p≤0.05, ** p≤0.001, *** p≤0.0001.

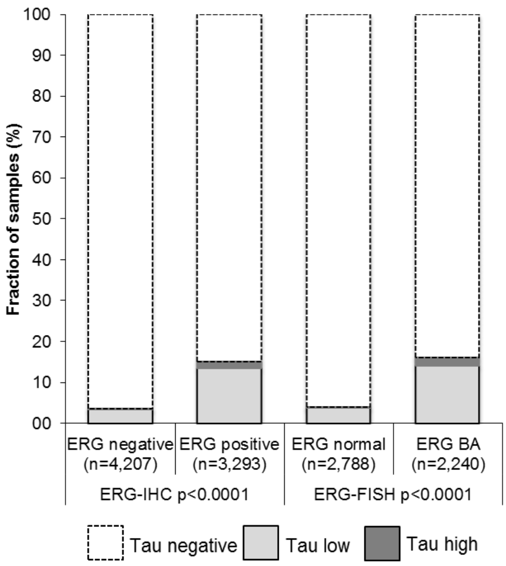

Fig. S1 Association between positive microtubule-associated protein Tau (MAPT) staining and ERG status (IHC/FISH) in all cancers.
